# Nanoscale Iron Redistribution during Thermochemical Decomposition of CaTi_1–*x*_Fe_*x*_O_3−δ_ Alters the Electrical Transport Pathway: Implications for Oxygen-Transport Membranes, Electrocatalysis, and Photocatalysis

**DOI:** 10.1021/acsanm.2c04537

**Published:** 2023-01-20

**Authors:** Jason Luong, Xin Wang, Alicia Tsung, Nicholas Humphrey, Huiming Guo, Benjamin X. Lam, Shaama Mallikarjun Sharada, William J. Bowman

**Affiliations:** † Department of Materials Science and Engineering, University of California, Irvine, Irvine, California92697, United States; ‡ Mork Family Department of Chemical Engineering and Materials Science, University of Southern California, Los Angeles, California90089, United States; § Department of Chemistry, University of Southern California, Los Angeles, California90089, United States; ∥ Irvine Materials Research Institute, University of California, Irvine, Irvine, California92697, United States

**Keywords:** oxide, perovskite, ceramic, earth-abundant, thermochemical reduction, decomposition, scanning transmission electron microscopy, electrical conductivity

## Abstract

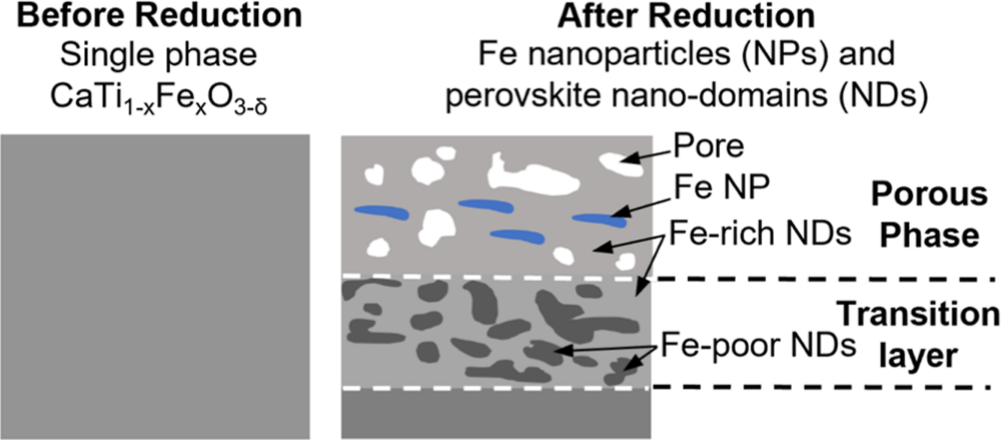

Potential applications of the earth-abundant, low-cost, and non-critical perovskite CaTi_1–*x*_Fe_*x*_O_3−δ_ in electrocatalysis, photocatalysis, and oxygen-transport membranes have motivated research to tune its chemical composition and morphology. However, investigations on the decomposition mechanism(s) of CaTi_1–*x*_Fe_*x*_O_3−δ_ under thermochemically reducing conditions are limited, and direct evidence of the nano- and atomic-level decomposition process is not available in the literature. In this work, the phase evolution of CaTi_1–*x*_Fe_*x*_O_3−δ_ (*x* = 0–0.4) was investigated in a H_2_-containing atmosphere after heat treatments up to 600 °C. The results show that CaTi_1–*x*_Fe_*x*_O_3−δ_ maintained a stable perovskite phase at low Fe contents while exhibiting a phase decomposition to Fe/Fe oxide nanoparticles as the Fe content increases. In CaTi_0.7_Fe_0.3_O_3−δ_ and CaTi_0.6_Fe_0.4_O_3−δ_, the phase evolution to Fe/Fe oxide was greatly influenced by the temperature: Only temperatures of 300 °C and greater facilitated phase evolution. Fully coherent Fe-rich and Fe-depleted perovskite nanodomains were observed directly by atomic-resolution scanning transmission electron microscopy. Prior evidence for such nanodomain formation was not found, and it is thought to result from a near-surface Kirkendall-like phenomenon caused by Fe migration in the absence of Ca and Ti co-migration. Density functional theory simulations of Fe-doped bulk models reveal that Fe in an octahedral interstitial site is energetically more favorable than in a tetrahedral site. In addition to coherent nanodomains, agglomerated Fe/Fe oxide nanoparticles formed on the ceramic surface during decomposition, which altered the electrical transport mechanism. From temperature-dependent electrical conductivity measurements, it was found that heat treatment and phase decomposition change the transport mechanism from thermally activated p-type electronic conductivity through the perovskite to electronic conduction through the iron oxide formed by thermochemical decomposition. This understanding will be useful to those who are developing or employing this and similar earth-abundant functional perovskites for use under reducing conditions, at elevated temperatures, and when designing materials syntheses and processes.

## 1 Introduction

The earth-abundant, low-cost, and non-critical perovskite CaTiO_3_ has been studied for its mechanical, optical, electrical, magnetic, and photocatalytic properties and for applications in oxygen-transport membranes (OTMs).^1−9^ OTM materials are mixed oxygen ion- and electron-conducting solids that enable technologies for water splitting, value-added chemical production, oxyfuel combustion, and pre-combustion CO_2_ separation, but functional membrane materials decompose under high oxygen chemical potential gradients.^10−13^ Remarkably, even though the maximum oxygen permeation performance is inferior to state-of-the-art iron-cobaltite oxides, the oxygen flux of CaTi_0.9_Fe_0.1_O_3−δ_ was shown to be more stable during a semi-permeation experiment in a simulated service atmosphere of CO, CO_2_, H_2_, and CH_4_, where no evidence of membrane decomposition or reaction byproducts was found after 1600 h.^8^ This demonstrated the potential of Fe-doped CaTiO_3_ (CaTi_1–*x*_Fe_*x*_O_3−δ_) as a more sustainable and non-critical material for OTM applications.

Considering the potential of OTM technologies to contribute to CO_2_ utilization, CaTi_1–*x*_Fe_*x*_O_3−δ_ has attracted attention due to its durability in the presence of CO_2_. Introducing Fe and other transition metals as solutes in CaTiO_3_ improves ionic and electronic conductivity^4,7,14−17^ and long-term stability due to its chemical and mechanical properties.^8,9^ In addition to optimizing the chemical composition, ceramic morphology control has proven useful: Adding porous CaTi_1–*x*_Fe_*x*_O_3−δ_ layers atop a dense membrane increases the active surface area and facilitates the impregnation of metal nanoparticles which increases oxygen permeability.^14^ However, CaTi_1–*x*_Fe_*x*_O_3−δ_ has also been shown to undergo thermal decomposition in H_2_ at elevated temperatures, forming metallic iron; such decomposition of OTM device components could affect surface phase stability and the oxygen surface exchange process that limits the overall oxygen flux.^14,18^ Thus, progress needs to be made to understand CaTi_1–*x*_Fe_*x*_O_3−δ_ phase stability in thermochemical applications,^13^ particularly at the unexplored atomic- and nanoscales and in porous ceramics.

Although it may challenge the implementation of CaTi_1–*x*_Fe_*x*_O_3−δ_ in OTMs, chemical instability presents an exciting opportunity in other contexts. For instance, the process of cation exsolution—which is akin to oxide phase decomposition—provides a finely tunable in situ synthesis route to durable metal nanoparticle catalysts embedded in the surface of various oxide supports.^19−25^ Perovskite oxides readily incorporate transition metals into the lattice during material synthesis under oxidizing conditions; when subsequently exposed to reducing conditions, the oxide undergoes a controllable phase decomposition, and transition metals can be selectively exsolved as dispersed catalytically active nanoparticles. The exsolution method is increasingly popular as it can overcome typical drawbacks—including nanoparticle agglomeration and deactivation—which are typical of traditional nanoparticle catalyst synthesis methods such as impregnation or vapor deposition. Very recently, it was found that Fe exsolution from SrTi_0.65_Fe_0.35_O_3_ thin films results in an Fe-depleted surface layer of ∼2 nm thickness.^26^ To further develop CaTi_1–*x*_Fe_*x*_O_3−δ_ as an earth-abundant catalyst support, exsolution of Fe (and/or other metals) could enable better catalytic activity and stability. It is thus useful to determine the critical temperatures and phenomena which occur during decomposition of the parent perovskite.

The tendency and mechanisms of CaTi_1–*x*_Fe_*x*_O_3−δ_ perovskites to decompose in reducing atmospheres at elevated temperatures is not well understood down to the atomic and nanoscale. Moreover, improved understanding of this process offers the potential to design advanced exsolution syntheses in this promising earth-abundant and non-critical perovskite. Defect chemistry studies have been performed on dense CaTi_1–*x*_Fe_*x*_O_3−δ_ ceramics down to very low oxygen partial pressures (pO_2_) and at temperatures around 1000 °C, assuming chemical phase stability. However, there are indications that this compound may not be stable in the absence of oxygen permeation flux, at which point Fe metal has been observed—presumably resulting from oxide decomposition. It is thus important to understand the decomposition mechanism and relevant processing conditions.

Here, we have done this in an unprecedented way by combining multiscale characterization down to the atomic scale, coupled with density functional theory (DFT) calculations, and electrical conductivity measurements over a range of chemical compositions and thermochemical heat-treatment conditions. We fabricated porous perovskite CaTi_1–*x*_Fe_*x*_O_3−δ_ ceramics with varied Fe B-site substitution and investigated the effects of thermochemical reduction by H_2_ diluted in Ar. We demonstrated variable phase evolution and decomposition of CaTi_1–*x*_Fe_*x*_O_3−δ_ over a range of temperatures and Fe doping amounts. Fully coherent Fe-rich and Fe-depleted perovskite nanodomains were, to our knowledge, observed directly for the first time by atomic-resolution scanning transmission electron microscopy (STEM) imaging and spectroscopy. Surprisingly, atomic-resolution elemental mapping by energy-dispersive X-ray spectroscopy (EDX) revealed that Fe occupies interstitial sites in the Fe-rich nanodomains. DFT simulations were employed to identify the energetically favorable sites for these Fe interstitials. Along with the nanodomains, reduction yielded agglomerated Fe/Fe oxide nanoparticles on the ceramic surface, which proved consequential to the electrical transport mechanism. Electrical conductivity measurements by electrochemical impedance spectroscopy (EIS) demonstrated the effect of both Fe doping and thermochemical decomposition on the overall transport mechanism of CaTi_1–*x*_Fe_*x*_O_3−δ_, which was altered by nanoscale redistribution of Fe into Fe/Fe-oxide phases at the ceramic surface. Ultimately, correlating these experimental and computational methods allowed us to identify the critical Fe contents and temperatures of phase decomposition, the influence of decomposition on electrical transport, and to propose a multi-length-scale decomposition mechanism down to the atomic level.

## 2 Methods

**Figure 1:**
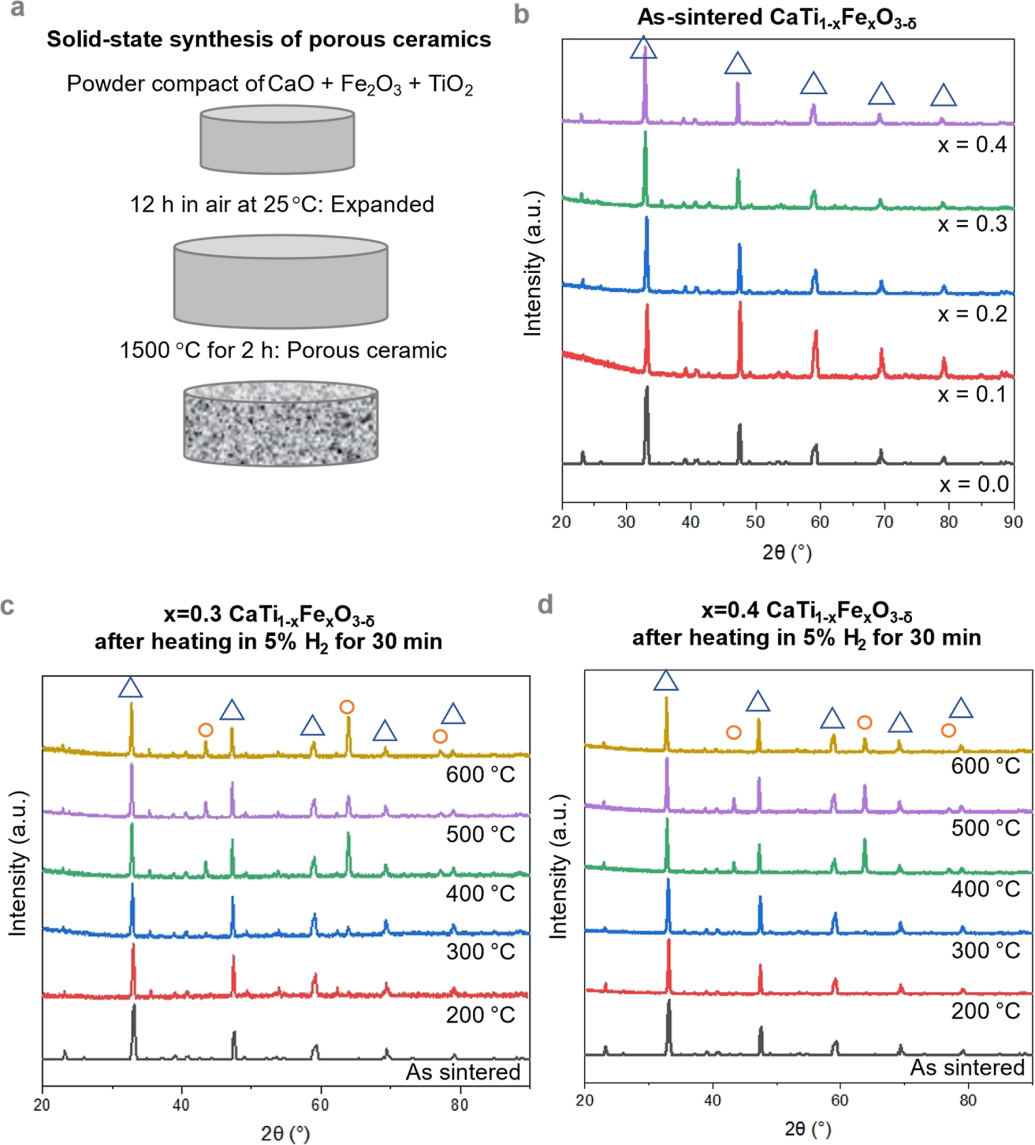
(a) Solid-state synthesis of porous ceramics was facilitated by the expansion of CaO upon reaction with H_2_O and/or CO_2_ in air prior to sintering. (b) XRD confirmed that the orthorhombic perovskite-type structure was obtained for all CaTi_1–*x*_Fe_*x*_O_3_ samples where 0 ≤ *x* ≤ 0.4 (blue triangles). (c,d) Thermochemical reduction of the samples *x* = 0.3 and *x* = 0.4 caused secondary Fe/Fe oxide phases to emerge at elevated temperatures (orange circles), as quantified in Table 1 and detailed in Figure S1.

**Figure 2:**
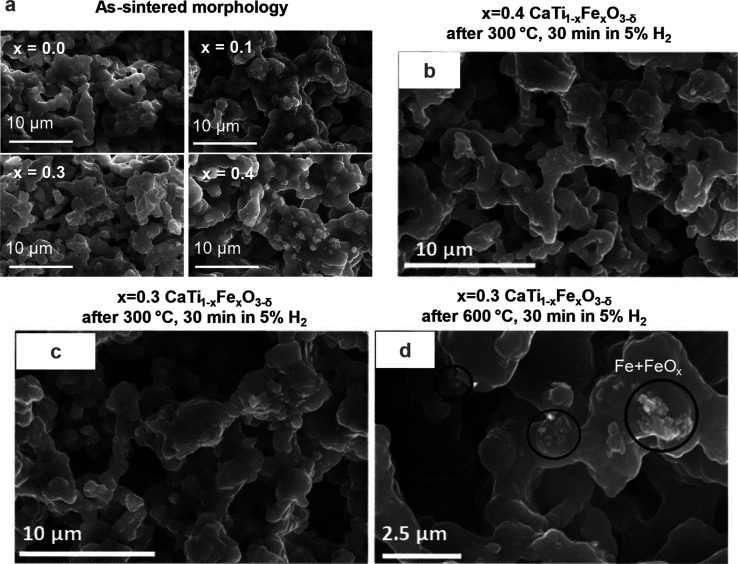
(a) As-sintered ceramics’ high porosity was confirmed by SEM SE imaging. (b–d) Porosity was maintained after thermochemical treatments of *x* = 0.4 after 300 °C (b), *x* = 0.3 after 300 °C (c), and *x* = 0.3 after 600 °C (d). Circles in (d) indicate the location of Fe/Fe-oxide phases formed on the ceramic surface, according to SEM-EDX and STEM analyses presented below.

Porous CaTi_1–*x*_Fe_*x*_O_3−δ_ was synthesized by solid-state reaction of CaO, TiO_2_, and Fe_2_O_3_ at 1500 °C for 2 h with a heating rate of 5 °C (Figure 1a) following ref.^27^ Stoichiometric amounts of precursor powders with 0 ≤ *x* ≤ 0.4 were mixed and ground by mortar and pestle and then pressed into pellets. Before sintering, the CaO in the pellets was allowed to react with air, likely forming a combination of Ca(OH)_2_ and CaCO_3_, which caused the green body to increase in volume (Figure 1a) and ultimately introduced extensive porosity in the sintered ceramic (Figure 2).

Thermochemical reduction of samples 0.1 ≤ *x* ≤ 0.4 was performed in a quartz tube under flowing 5% H_2_ in Ar at 200–600 °C for 30 min within a Lindberg tube furnace. The samples were heated to each treatment temperature in air, then at temperature the reducing gas was flowed for 30 min before the samples were cooled in air to room temperature.

The crystal structure and phase compositions were measured before and after thermochemical processing using an Ultima III X-ray diffractometer with Cu K_α_ radiation under 40 kV and 30 mA. The scanned range was 2θ = 20–90° with a step size of 2θ = 0.05°. The morphology and elemental distribution were investigated using a Magellan SEM at 25 kV and 25 pA equipped with an EDX spectrometer.

STEM was used to study the micro- and nano-scale morphology, as well as the atomic structure and elemental distribution in CaTi_0.6_Fe_0.4_O_3−δ_ after thermochemical processing at 600 °C, as this sample showed severe decomposition during heat treatment. To prepare the samples for STEM, focused ion beam (FIB) lift-out was performed on an FEI Quanta 3d dual-beam SEM system. An ∼200 nm thick Pt protection layer was first deposited on the region of interest with the electron beam at 5 kV, followed by a ∼4 μm ion beam deposition of Pt at 30 kV. To reduce the damage induced by the Ga ion beam, decreasing ion beam currents from 1 nA to 300 pA were used for the thinning steps. A final cleaning step was performed at 5 kV and 16 pA for 5 min on each side of the specimen. STEM characterization was carried out on a double aberration-corrected JEOL-ARM300F Grand ARM operated at 300 kV, equipped with double X-ray detectors for EDX elemental mapping.

Temperature-dependent EIS was used to measure the resistance of selected samples in air, which was then used to calculate the conductivity. Silver ink (Fuel Cell Materials) was coated on opposite sides of samples as porous electrodes. After coating, the samples were calcined at 100 and 800 °C in air for 1 h for drying and firing the silver paste, respectively (Carbolite Gero CC-T1). EIS tests were conducted using a potentiostatic mode with a frequency range of 0.1 Hz to 1 MHz (Gamry 1010E) connected to an electrochemical workstation (Huber Scientific, Plug and Probe) inserted into a tube furnace (Carbolite Gero CC-T1). During the EIS test, samples were heated from 25 to 425 °C in increments of approximately 50 °C for Arrhenius analysis. These temperatures were recorded with a thermocouple and used in Arrhenius fitting. About 10–15 EIS curves were measured at each temperature and averaged before being fitted to a resistor-constant phase element equivalent circuit model (“1RQ”).^28^ Selected samples were fitted to equivalent circuit models comprising series combinations of 1RQ subcircuits (“2RQ”), or 1RQ in series with a resistor, to improve the fitting if there were multiple arcs visible. The highest frequency arc was interpreted as the sample’s impedance contribution.

To interpret the atomic-resolution STEM-EDX of the Fe cation distribution observed in CaTi_0.6_Fe_0.4_O_3−δ_, DFT calculations were performed. All spin-polarized calculations were performed using the Vienna ab initio simulation package, VASP 5.4.^29−32^ The strongly constrained and appropriately normed (SCAN) meta-generalized gradient approximation (meta-GGA) was employed.^33^ The Atomic Simulation Environment (ASE) was utilized for model construction.^34^ All atoms were described using the default projector augmented wave (PAW) potentials available in VASP.^35,36^ Non-spherical contributions related to density gradient in the PAW spheres were included in the calculations, with d-orbitals included in kinetic energy density mixing. Bulk calculations were carried out using Γ-centered (9 × 9 × 9) *k*-point density. Gaussian smearing of 0.05 eV was used along with 400 eV cutoff energy. Lattice optimizations were carried out to determine bulk lattice parameters using a single cubic unit cell for CaTiO_3_. To approximately represent the Fe-doped system while keeping computations tractable, the unit cell was doubled and Fe was placed at either the tetrahedral or the octahedral interstitial site, along similar lines of a previous study with BaZrO_3_.^37^

## 3 Results and Discussion

### 3.1 Crystal Structure, Microstructure, and Morphology Analyses by XRD and SEM

XRD patterns of all as-sintered CaTi_1–*x*_Fe_*x*_O_3−δ_ samples were indexed to the single-phase CaTiO_3_ orthorhombic perovskite according to the ICDD database no. 00-042-0423 (Figures 1b and S1). The absence of additional Fe-containing phases confirms the compound’s high-temperature phase stability in air when substituting Ti with Fe, consistent with past work on this solid solution^5^ and the fact that Fe^4+^ and Ti^4+^ have similar ionic radii.^7^ Thermochemical heat treatments under flowing 5% H_2_/Ar resulted in the formation of additional Fe-containing crystalline phases at 200 and 300 °C in *x* = 0.3 and *x* = 0.4, respectively, which were found to coexist with the initial CaTiO_3_ perovskite-type structure by XRD (Figures 1c,d and S1). No Fe-containing crystalline phases were detected in the *x* = 0–0.2 samples, which remained entirely the CaTiO_3_ perovskite-type structure and were thus not further investigated here. The phase percentage of the perovskite CaTiO_3_ was calculated using the XRD peak intensity for the peaks Fe{200}/CTO{200}, Table 1. The estimation assumes that the peak intensity ratio equals the phase concentration ratio, and the phase concentrations sum to 1:  and *C*_FeO_*x*__ + *C*_CTO_=1.

**Table 1:** Evolution of Fe and CTO Phases after Heating in H_2_^a^

	CaTi_1–*x*_FexO_3−δ_	300 °C	400 °C	500 °C	600 °C
Fe{200}/CaTiO_3_{200}	*x* = 0.3	0.2	0.8	0.4	0.7
	*x* = 0.4		0.4	0.35	0.2
CaTiO_3_phase %	*x* = 0.3	83	56	71	59
	*x* = 0.4		71	74	83

a Tabulated are the ratios of XRD peak intensity for Fe{200}/CTO{200}. By quantifying the XRD peak intensity ratios of Fe{200}/CaTiO_3_{200}, it is apparent that the secondary phases emerged above 200 °C in *x* = 0.3 and above 300 °C in *x* = 0.4 (see also Figures S1d and S2d).

A close inspection of the perovskite matrix {200} peaks in *x* = 0.3 and *x* = 0.4 revealed a slight peak shift toward smaller Bragg angles with increasing heat-treatment temperature (Figures S1b and S2b). We estimated that this lattice expansion increased the unit cell volume of *x* = 0.3 and *x* = 0.4 from 0.161 to 0.166 nm^3^ (+3%) and 0.167 nm^3^ (+3.5%), respectively (Table S1). Expansion of the perovskite matrix can be attributed to an increased amount of Fe interstitial defects—for which we present direct experimental evidence below. Additionally, the ionic radii for 6-fold coordinated high-spin Fe^4+^, Fe^3+^, and Fe^2+^ are 0.585, 0.645, and 0.78 Å, respectively.^38^ Although we do not have oxidation state experimental data for Fe, this too could contribute to the observed lattice expansion. It is not understood why the *x* = 0.4 sample shows a peak in the amount of Fe/Fe oxide after thermochemical reduction at 400 °C.

The as-sintered porous ceramics had similar morphology according to SEM secondary electron (SE) imaging, Figure 2a. The as-sintered *x* = 0.4 porous ceramic surface is covered by small, ∼1 μm, grains, which are assumed to be perovskite based on phase-pure XRD data, Figure 1b,d. For this and all samples, we attempted SEM-EDX to map coarse surface agglomerates suspected to be Fe-rich but could not differentiate between Fe in surface agglomerates and Fe in the sub-surface perovskite matrix. It is unclear why these small grains formed on the *x* = 0.4 surface, though we speculate that the relatively high Fe content could contribute to solute drag effects which limit the incorporation of small grains into the porous superstructure. The morphology of the continuous solid shows negligible difference between the *x* = 0.3 and *x* = 0.4 at the initial stage of decomposition (after reduction at 300 °C) from SEM SE imaging (Figure 2b,c). In line with our XRD result, at later stages of decomposition, there was visible evidence in SEM SE images and STEM-EDX mapping (shown below) of morphology changes as a function of treatment temperature, such as the increased formation of surface features attributed to Fe metal and Fe oxide crystalline phases (e.g., *x* = 0.4 after 600 °C, Figure 2d).

While our results on porous *x* = 0.3 and *x* = 0.4 ceramics are qualitatively consistent with findings by Salles et al.—who found that a dense CaTi_0.9_Fe_0.1_O_3−δ_ ceramic underwent minor decomposition to Fe metal during thermochemical reduction at 850 °C^8^—our samples underwent severe secondary phase formation at just 200–300 °C. Salles et al. observed partial reduction of Ti-substituting Fe^4+^ to Fe^3+^ (Fe_Ti_^/^) under H_2_ at 350 °C via H_2_ oxidation by lattice oxygen, followed by the formation of metallic Fe crystallites (visible by XRD) at 850 °C. Here, we hypothesize that the lower decomposition temperature is a combined effect of (i) higher Fe concentration and (ii) more surface area in our porous samples: the greater surface area facilitates oxygen vacancy (V_O_^••^) formation at the solid surface even at the low temperatures used in our study, which then electrostatically attract reduced Fe cations (Fe_Ti_^/^ and Fe_Ti_^//^) to the surface. Fe migration to regions of higher oxygen vacancy concentration is likely driven by their negative binding energy with oxygen vacancies Fe_Ti_^/^–V_O_^••^ in dimer and trimer defect clusters.^7^ That we observe Fe metal and oxides via decomposition at significantly lower temperatures than Salles et al.’s CaTi_0.9_Fe_0.1_O_3−δ_ suggests that there is a balance between increased ceramic surface area, Fe concentration, and thermochemical stability.

### 3.2 Nanoscale Morphology and Elemental Distribution after Decomposition of CaTi_0.6_Fe_0.4_O_3−δ_

**Figure 3:**
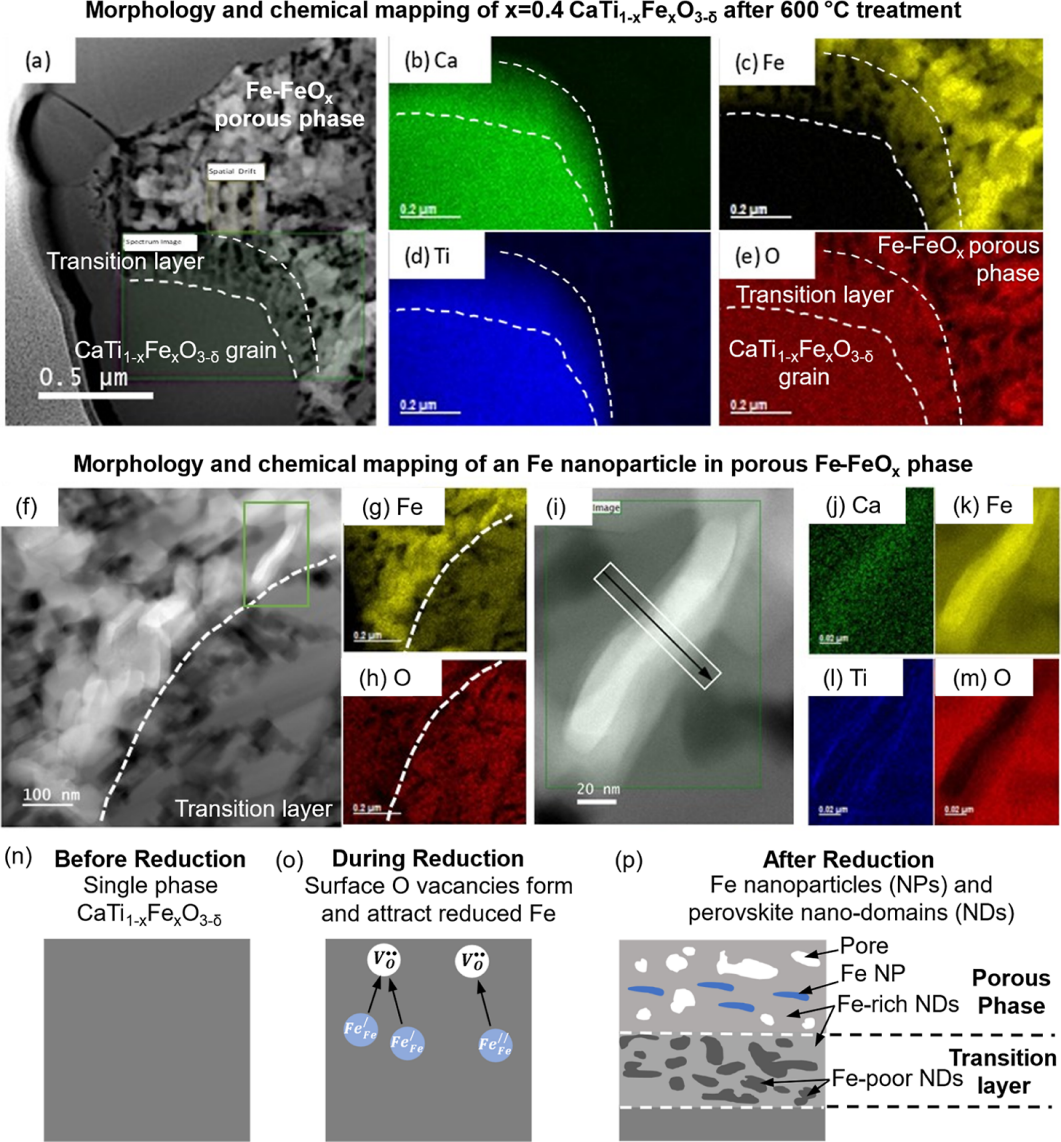
Nanoscale morphology and chemical analysis of CaTi_0.6_Fe_0.4_O_3−δ_ after thermochemical treatment at 600 °C revealed three distinct phases. (a) STEM-HAADF image near the ceramic surface (the FIB Pt protection layer is at the far left of the image and appears bright); the dashed lines indicate the borders between (1) the CaTi_1–*x*_Fe_*x*_O_3_ grain and the transition layer that contains Fe-rich and Fe-poor nanodomains and (2) the transition layer and the porous phase containing Fe metal and Fe oxide nanoparticles. (b–e) EDX maps for Ca, Fe, Ti, and O, respectively, of the region marked by a green box in (a). (f–h) STEM-HAADF image with Fe and O EDX maps of the border (dashed line) between the porous Fe/Fe-oxide nanoparticles and the transition layer. (i–m) STEM-HAADF image and EDX maps of an Fe nanoparticle in the boxed region in (f); see also Figure S3 for the line profiles corresponding to the black arrow in (i). (n–p) Proposed decomposition mechanism of *x* = 0.3 and *x* = 0.4 CaTi_1–*x*_Fe_*x*_O_3_ forms a coherent transition layer of Fe-rich and Fe-poor nanodomains and a porous phase of Fe metal and Fe oxide nanoparticles.

To further understand the decomposition mechanism, STEM high-angle annular dark field (HAADF) imaging and EDX spectroscopy were used to analyze the nanoscale morphology and elemental distribution in the *x* = 0.4 sample after reduction by H_2_ at 600 °C. This sample contained three noteworthy phases: Fe-deficient CaTi_0.6_Fe_0.4_O_3−δ_ perovskite grains, a so-called “transition layer” containing Fe-rich and Fe-poor nanodomains which are coherent with the perovskite, and a nanoporous agglomerate of intermixed Fe-metal and Fe-oxide nanoparticles (Figure 3). STEM-EDX elemental maps indicate that the remaining grains of the perovskite ceramic contain primarily Ca, Ti, and O and are depleted of Fe—which apparently migrated to the oxide’s surface during decomposition (Figure 3a–e). Surprisingly, on the surface of the perovskite grain, there is a ∼200 nm thick transition layer (outlined by white dashed lines in Figure 3a–e) with a decreasing concentration gradient of Ca and Ti and an increasing concentration gradient of Fe. Within the transition layer, there is a non-uniform distribution of Fe, with so-called “Fe-rich nanodomains” and “Fe-poor nanodomains” appearing bright and dark, respectively, in both Fe EDX maps (Figure 3c,g) and HAADF images (Figure 3a,f). Opposite to the transition layer from the perovskite grains is the nanoporous phase comprising an intimate mixture of agglomerated Fe metal and Fe oxide nanoparticles that is strongly/entirely absent of Ca and Ti (Figure 3f–m). The Fe nanoparticles appear bright in the HAADF-STEM images (e.g., Figure 3i) and EDX maps (Figure 3k) due to the local enrichment of the heavier Fe atoms, while nanopores appear as dark areas in the HAADF image and Fe and O EDX maps. The complexity of the Fe nanoparticles likely warrants further investigation beyond this study; for instance, EDX of an individual Fe nanoparticle also revealed trace amounts of Ca in the particle and Ti accumulation on the particle surface perhaps because Fe and Ti form the stable ilmenite perovskite FeTiO_3_.

The proposed nanoscale phase decomposition mechanism observed in the *x* = 0.3 and *x* = 0.4 samples is summarized as follows (Figure 3n–p): (1) high-temperature annealing in air during porous ceramic synthesis yields a single-phase perovskite solid solution with a negligibly low concentration of oxygen vacancies compensating reduced Fe and Ti, which are randomly distributed.^39^ (2) The H_2_ thermochemical reduction environment reduces the pO_2_ such that Fe metal is in thermodynamic equilibrium with the oxide.^8,40,41^ Additionally, reduction by H_2_ creates lattice oxygen vacancies (V_O_^••^) near the ceramic’s surface that are charge compensated by Fe reduction from 4+ to 3+ and/or 2+ (Fe_Ti_^/^ and/or Fe_Ti_^//^)^8^ like the atomistic exsolution process used to synthesize surface nanostructures in situ. The high concentration of near-surface oxygen vacancies contributes an electrostatic driving force for migration reduced Fe cations through the perovskite toward the surface where they are reduced to Fe metal,^7,22^ albeit at unexpectedly low temperatures for cation diffusion. (3) The Fe diffusing out of the perovskite is believed to originate in the transition layer, a volume of perovskite lattice that is both coherent with the underlying grain and riddled with Fe-poor nanodomains after decomposition. Although cation substitutional diffusion would be the expected Fe transport mechanism toward the surface in single-phase perovskite,^7^ this is apparently not entirely the case here. As supported by direct atomic-resolution EDX maps and DFT calculations presented below, Fe apparently migrates out of the transition layer to the Fe metal/Fe oxide porous layer in part via interstitial diffusion in Fe-rich nanodomains observed in the transition layer.

### 3.3 Atomic Structure and Elemental Distribution of the Transition Layer and Nanodomains in CaTi_0.6_Fe_0.4_O_3−δ_

**Figure 4:**
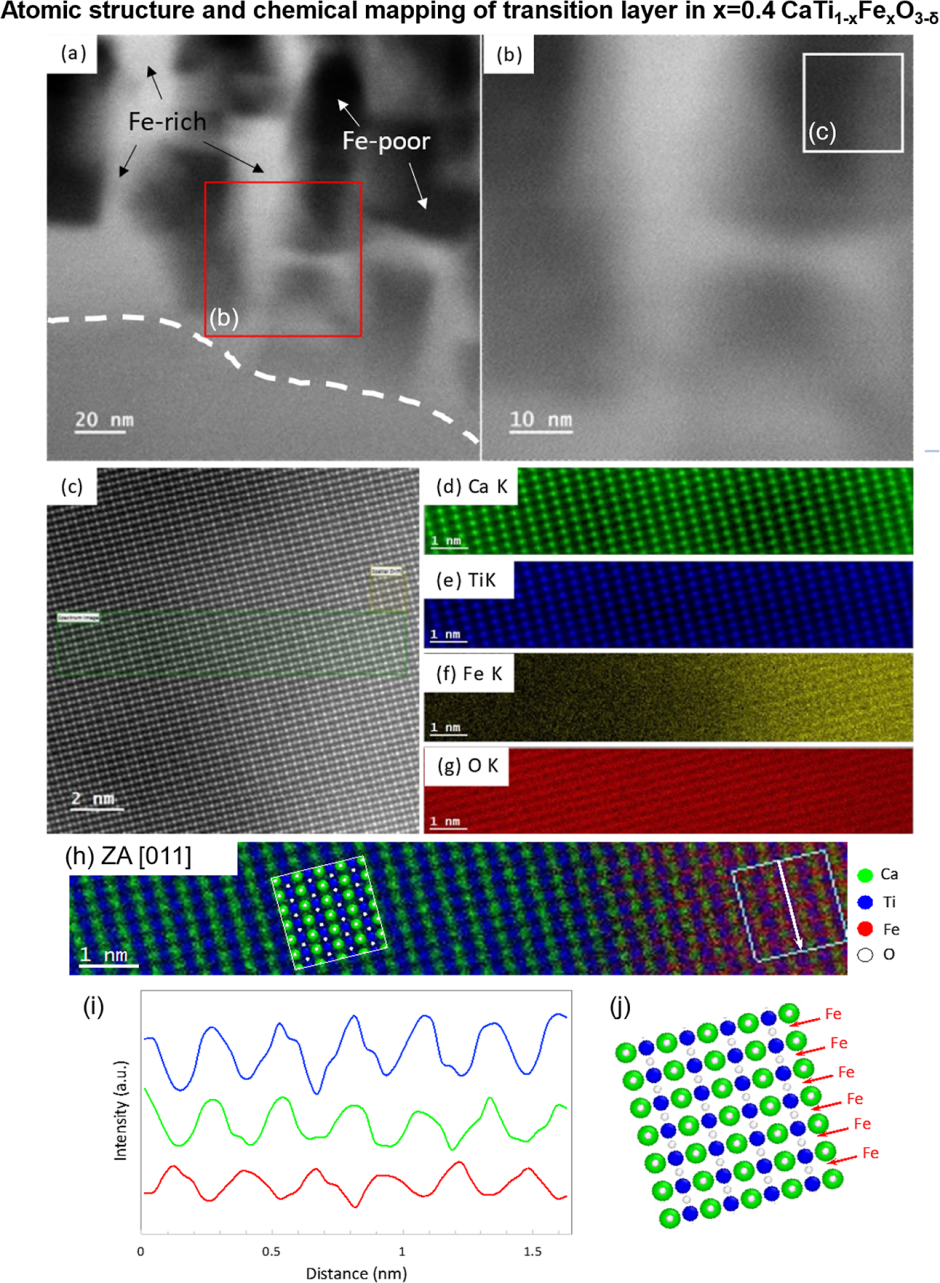
Transition layer in CaTi_0.6_Fe_0.4_O_3−δ_ is coherent with the perovskite grain, with Fe^2+^ interstitials as octahedral sites. (a,b) STEM-HAADF images showing the Fe-rich and Fe-poor nanodomains in the transition layer with average intervals of around 20 nm. The dashed line is the border between the CaTi_0.6_Fe_0.4_O_3−δ_ grain and the transition layer. (c) Atomic-resolution STEM-HAADF image taken at the region marked by a white box in (b) along the [011] direction. (d–g) STEM-EDX maps for Ca, Ti, Fe, and O, respectively, of the region marked by a green box in (c), at the interface between Fe-poor [left side of (f)] and Fe-rich [right side of (f)] nanodomains. (h–j) Composite EDX cation maps (d–g), EDX signal line scan along the white arrow depicted in (h) and averaged over the width of the box, and perovskite crystal model indicating Fe occupying sites on the Fe–O plane.

Given our unexpected discovery of the transition layer, atomic-resolution STEM imaging and EDX coupled with DFT calculations were used to conclude that Fe in the Fe-rich nanodomains exists as Fe^2+^ interstitials, which are energetically most favorable in octahedral sites (Figure 4). The transition layer borders the perovskite CaTi_0.6_Fe_0.4_O_3−δ_ matrix and consists of atomically coherent Fe-rich and Fe-poor nanodomains 20–100 nm in size (Figure 4a–c). Fe-rich and -poor nanodomains show brighter and darker HAADF contrast, respectively, caused by different Fe contents, as confirmed by EDX (Figure 4f). Atomic-resolution STEM-EDX confirms that Ca, Ti, and O ions exist in both types of nanodomains. To our knowledge, this is the first observation of such structurally coherent nanodomains formed during perovskite oxide decomposition. Nanodomain formation was not observed in the perovskite bulk and so is assumed to be associated with the near-surface decomposition process. A possible nanodomain formation mechanism is related to the nanoscale Kirkendall effect, where in this case Fe migration from the perovskite to the surface proceeds faster than the reverse process, thereby depleting the transition layer of Fe and creating the Fe-poor nanodomains. However, rather than creating Kirkendall voids (e.g., as seen in NiO^42^), Fe-poor nanodomains form in a stable CaTiO_3_-like perovskite because Fe is the only cation diffusing from the transition layer to the surface.

Surprisingly, a closer inspection of the composite cation EDX maps indicated that Fe is located at cation interstitial sites in the Fe-rich nanodomains, suggesting local interstitial Fe diffusion (Figures 4d–j and S5). The possibility that this observation is caused by projection through the perovskite and an additional Fe-rich oxide particle is precluded by the fact that the Ca/O and Ti/O EDX signal intensity ratios remain constant across the Fe-rich/Fe-poor phase boundary in the analyzed area. If the STEM specimen contained an additional Fe-rich oxide particle overlapping the perovskite, instead of Fe interstitials, the Ca/O and Ti/O would decrease in the area occupied by the Fe-rich oxide particle because the contribution of Ca and Ti to the total cation signal would necessarily decrease, while the anion signal would remain approximately uniform. This direct spectroscopic evidence of Fe between two {0-11} planes of alternating Ca and Ti atoms indicates that Fe is located on the same perovskite lattice planes as the O sites within the Fe-rich nanodomain (red arrows in Figure 4j). This means that Fe occupies either octahedral or tetrahedral interstitial sites in the perovskite lattice; we calculate the relative energies of these Fe interstitial defects using DFT below. This is quite surprising, given that cation interstitials and Frenkel disorder in the close-packed perovskite system are expected to be energetically unfavorable^43,44^ at equilibrium (e.g., >3.6 eV formation energy^7^). However, first-principles calculations by Polfus et al. have shown that because of their reduced ionic radius, divalent transition-metal cations (e.g., Ni^2+^) can dissolve into zirconate perovskite oxides with large lattice volumes by occupying the octahedral interstitial site that is square planar coordinated to four oxide ions on the cell edge face center.^37^ (Occupation of the tetrahedral interstitial site—which in our case would be coordinated to Ca and three oxide ions—was found to be unfavorable in ref.^37^) Thus, ours could be an observation of Fe^2+^ migration via octahedral interstitials or the system in a metastable state amenable to cation interstitials.

### 3.4 Determination of the Energetically Favorable Fe Interstitial Location by DFT

The surprising existence of interstitial Fe cations in the Fe-rich nanodomain is interpreted initially following the work of Polfus et al., who showed that Ni^2+^ interstitial solutes in BaZrO_3_ are more energetically favorable at octahedral sites than tetrahedral sites according to DFT.^37^ Following this approach and considering our direct experimental evidence of interstitial Fe, we employed DFT calculations to suggest which interstitial site—octahedral or tetrahedral—is the most energetically favorable for Fe^2+^ to occupy. We only considered Fe interstitials based on direct evidence from STEM-EDX mapping (Figure 4i,j), which are effective positive charges (e.g., Fe_i_^••^). We assumed charge compensation by Ti reduction from Ti^4+^ to Ti^3+^ (Ti_Ti_^/^) or Ti^2+^ (Ti_Ti_^//^), and that interstitial Fe was Fe^2+^ as this would require less total charge compensation than interstitial Fe^3+^.

**Figure 5:**
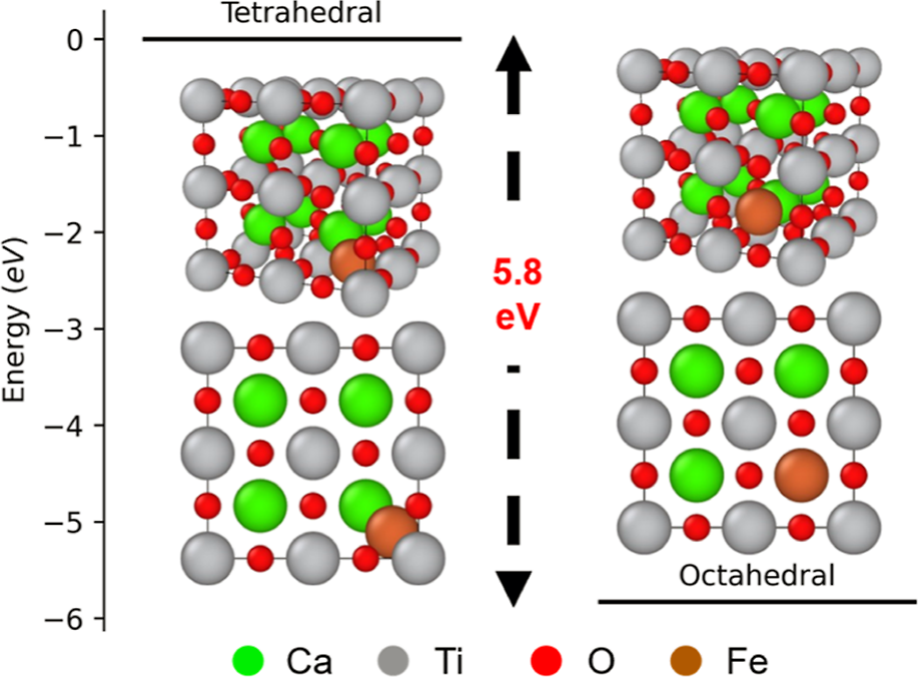
DFT-optimized structures of Fe atom at the tetrahedral (left) and octahedral (right) positions of CaTiO_3_. Fe at the octahedral site is 5.8 eV lower in energy relative to Fe at the tetrahedral site.

DFT simulations optimizing the bulk 2 × 2 × 2 CaTiO_3_ supercell showed that Fe occupying an octahedral interstitial site is 5.8 eV lower in energy than that in a tetrahedral interstitial site (Figure 5), in agreement with the work of Polfus et al. Fe at the tetrahedral site leads to uniform lattice expansion along all directions (lattice parameter increases from 7.726 Å in CaTiO_3_ to 7.822 Å upon Fe doping). Fe at the octahedral site distorts the cubic structure, leading to lattice contraction along two dimensions (7.673 Å) and expansion along the third (7.856 Å). Bader analysis is performed to assess charge compensation effects among cationic species. Fe is slightly more cationic at the tetrahedral site (+0.73 e) relative to the octahedral site (+0.58 e).^45,46^ However, it is difficult to draw meaningful conclusions regarding the mechanism of charge transfer. This is because changes in oxidation state are not always easy to discern from Bader analysis. In this study, for instance, the reduction in charges on Ca (from +1.63 e in CaTiO_3_ to +1.33 e for octahedral and +1.23 e for tetrahedral) and Ti (from +1.89 e in CaTiO_3_ to +1.91 e for octahedral and +1.63 for tetrahedral) in species that are closest to the Fe cation are both small.

### 3.5 Electrical Transport of CaTi_1–*x*_Fe_*x*_O_3−δ_ and the Effect of Thermochemical Decomposition

**Figure 6:**
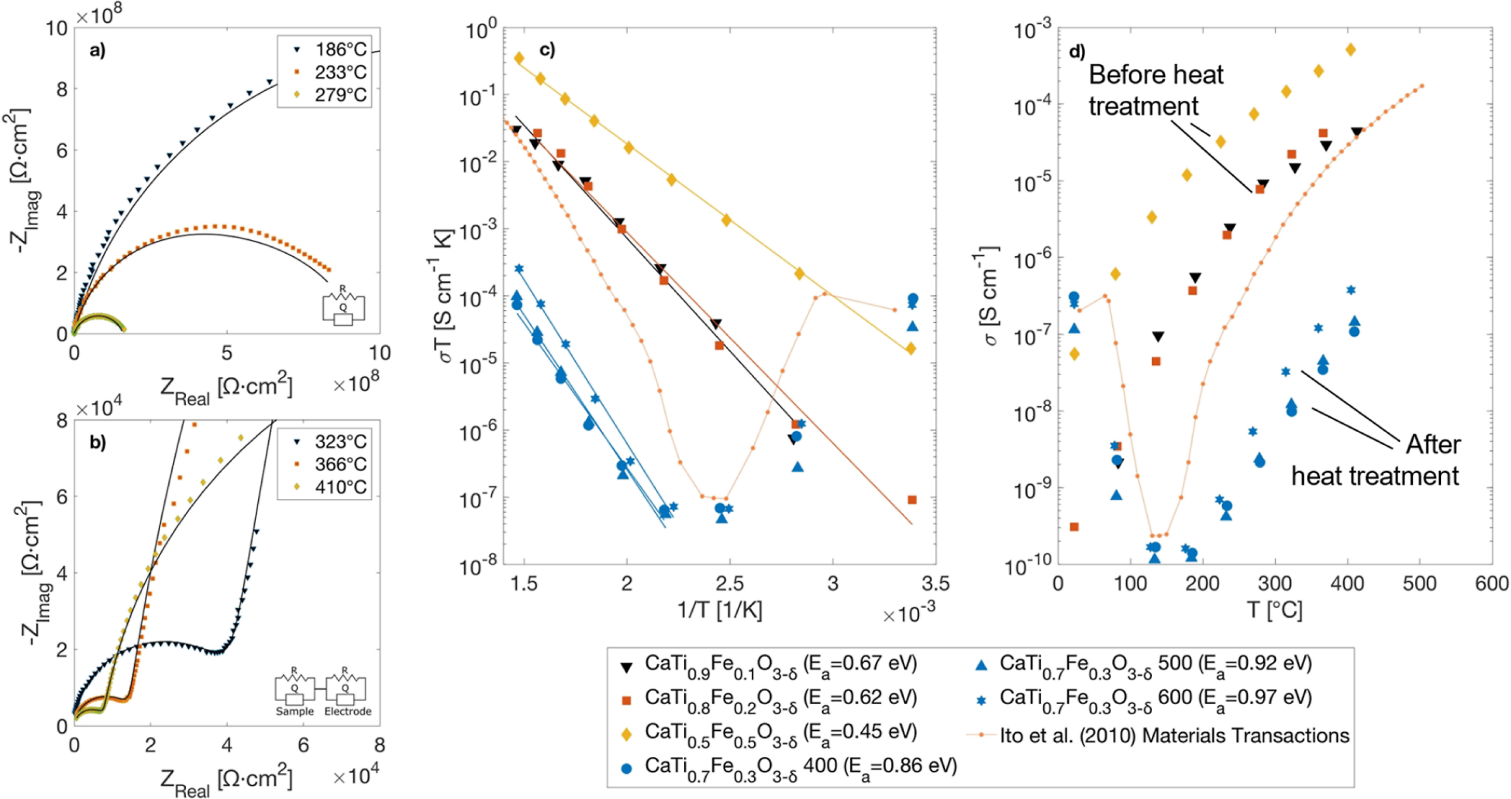
Electrical conductivity of select CaTi_1–*x*_Fe_*x*_O_3−δ_ samples before and after thermochemical decomposition showed a distinct change in the charge-transport mechanism. Representative EIS data acquired in air at various temperatures for (a) CaTi_0.7_Fe_0.3_O_3−δ_ following thermochemical treatment at 400 °C and for (b) CaTi_0.8_Fe_0.2_O_3−δ_ before thermochemical treatment. The solid lines in (a,b) are equivalent circuit models fitted to the measured data. The resistance values extracted from equivalent circuit modeling were converted to conductivities and plotted against temperature (d), which were fitted in Arrhenius plots to calculate the activation energies for conductivity (c). For the thermochemically treated samples (CaTi_0.7_Fe_0.3_O_3−δ_), the conductivity below and above 150 °C exhibits the opposite temperature dependence consistent with electron transport through Fe oxide reported by Ito et al.; reproduced with permission from ref (47).

Because the perovskites CaTiO_3_ and CaTi_1–*x*_Fe_*x*_O_3−δ_ are researched for applications relying on their electronic and mixed oxygen ionic and electronic transport (e.g., OTMs, photocatalysis, and electrocatalysis), we used temperature-dependent electrical measurements on select samples to both benchmark our materials and understand the effect of decomposition on transport properties (Figures 6 and S4). EIS was performed at low and intermediate temperatures (<400 °C) on the as-prepared CaTi_1–*x*_Fe_*x*_O_3−δ_ samples (*x* = 0.1, 0.2, and 0.5) before thermochemical treatment and on the CaTi_0.7_Fe_0.3_O_3−δ_ sample after thermochemical treatments at 400, 500, and 600 °C (Figure 6a,b). This provided a convenient way to compare our as-fabricated materials’ conductivity to that in the literature and to infer the effect of thermochemical treatment temperature and phase decomposition on the electrical properties. The EIS samples were prepared by applying liquid Ag paste to opposite sides of the ceramic pellets and then firing the sample at 800 °C in air to decompose all organics in the Ag ink solution, ultimately forming a porous Ag electrode. This firing procedure is also expected to oxidize any pure Fe metal particles (discussed above) in the heat-treated CaTi_0.7_Fe_0.3_O_3−δ_, yielding a porous ceramic composite of perovskite CaTi_1–*x*_Fe_*x*_O_3−δ_ and Fe oxide nanostructures.

As expected from the original publication by Iwahara et al.,^5^ and more recent work by Salles et al.^17^ and others,^15,16,48^ the conductivity of our as-prepared CaTi_1–*x*_Fe_*x*_O_3−δ_ samples (before thermochemical treatment) is attributed to thermally activated electron hole conduction in the measurement temperature range and pO_2_ of air. From Arrhenius plots of ln(σ*T*) versus 1/*T*, we measured conductivity activation energies of 0.45–0.67 eV (Figure 6c), which is 0.20–0.33 eV less than the reported activation energy for oxygen ionic conductivity in similar compositions of 0.87–1 eV (e.g., refs (4) and (17)). Also, like Iwahara et al., *x* = 0.4 showed the highest conductivity compared to both *x* = 0.1 and *x* = 0.2.

Interestingly, the combination of thermochemical treatment of CaTi_0.7_Fe_0.3_O_3−δ_ and EIS electrode fabrication reversed the temperature dependence of conductivity below ∼150 °C (Figure 6c,d) and increased the conductivity activation energy above ∼150 °C to 0.86–0.97 eV (Figure 6c). This is attributed to (1) the fact that thermochemical reduction heat treatment causes Fe and Fe oxide nanostructures to form on the perovskite surface and (2) that EIS electrode fabrication oxidizes the Fe. The conductivity that is therefore measured by EIS is electronic transport through the iron oxide formed during thermochemical treatment and/or during EIS electrode fabrication. Ito et al. have reported a very similar temperature dependence of the electrical conductivity of sintered α-Fe_2_O_3_, which showed a decrease in conductivity from 70 to 150 °C caused by phonon scattering that lowered the carrier mobility.^47^ At temperatures >150 °C, they measured the conductivity activation energy to be 1.05 eV,^47^ which is only slightly greater than that measured in CaTi_0.7_Fe_0.3_O_3−δ_ here. Thus, we attribute the conductivity after heat treatment to electron conductivity through iron oxide formed via decomposition by H_2_ and subsequent oxidation.

Alternative explanations for the transport behavior of *x* = 0.3 after heat treatments were deemed unlikely. First, below 150 °C, conductivity decreases with increasing temperature, which could be facilitated by band conduction through Fe metal, though this is unlikely considering that exposing the sample to air at 800 °C during preparation of the Ag EIS electrode should oxidize any metallic iron created during thermochemical treatments. Second, above 150 °C, conductivity increases with temperature, as expected for a thermally activated conduction mechanism. The conductivity activation energy calculated in this temperature regime ranges from 0.86 to 0.97 eV for 400 and 600 °C treatments, respectively, which could be interpreted as oxygen anion conduction through the perovskite, for example, 0.91 to 1.19 eV from ref.^16^ However, the electrolytic domain of CaTi_1–*x*_Fe_*x*_O_3−δ_ is typically reported to be below pO_2_ = 10^–7^ atm, which is several orders of magnitude lower than that of our air measurements and likely precludes oxygen conduction as a relevant transport mechanism in this study.

## 4 Conclusions

This study reveals unprecedented details about the nano- and atomic-level phase evolution of porous CaTi_1–*x*_Fe_*x*_O_3−δ_ ceramics caused by thermochemical reduction heat treatments and the implication of decomposition on electrical transport. This is a crucial property for applications including OTMs, electrocatalysis, and photocatalysis. By varying the amount of Fe in the system, we find that CaTi_1–*x*_Fe_*x*_O_3−δ_ undergoes a complex phase decomposition to agglomerated Fe/Fe oxide nanoparticles accompanied by the creation of a non-uniform transition layer near the surface in higher Fe-containing systems. XRD analysis after thermochemical treatments show secondary phases which were indexed to Fe/Fe oxide. SEM, STEM, and EDX revealed the agglomeration of Fe nanoparticles due to phase decomposition, which is facilitated by iron cation migration. Interestingly, Fe-rich and Fe-poor nanodomains were created in a transition layer between the single-phase perovskite and the porous nanostructured surface layer comprising agglomerated Fe and Fe oxide nanoparticles. This has been probed in the exemplary system CaTi_0.6_Fe_0.4_O_3−δ_ with atomic spatial resolution and DFT calculations, which together revealed that Fe is likely located at octahedral interstitials square planar coordinated by oxide ions in the Fe-rich nanodomains. Prior evidence for such nanodomain formation was not found and is thought to result from a near-surface Kirkendall-like phenomena caused by Fe migration in the absence of Ca and Ti migration. The effect of decomposition on the electrical properties of the samples was determined using EIS, which revealed that the charge-transport mechanism changes from thermally activated p-type conductivity through the perovskite to, most likely, electronic conduction through the iron oxide formed by thermochemical decomposition. The knowledge produced by this study is useful to readers who are interested in using electrochemical and functional perovskites under reducing conditions and/or at elevated temperatures and designing nanoscale materials via phase decomposition such as by the exsolution mechanism. While there is no evidence of submerged or partially submerged nanostructures resulting from the exsolution mechanism, the formation of agglomerated Fe nanoparticles and the Fe-poor/-rich nanodomains observed in this study expands our understanding of key aspects of the temperature-dependent thermochemical phase decomposition of CaTi_1–*x*_Fe_*x*_O_3−δ_.

## Supporting Information Available

XRD patterns and unit cell volume change of CaTi_0.7_Fe_0.3_O_3−δ_ and CaTi_0.6_Fe_0.4_O_3−δ_ after thermochemical treatments of 200–600 °C; EDX elemental signal profile along the line in Figure 3i; and representative EIS measurements for all samples across three temperatures (PDF)
