# Supplementary material for: Nanoscale Iron Redistribution during Thermochemical Decomposition of CaTi1–xFexO3−δ Alters the Electrical Transport Pathway: Implications for Oxygen-Transport Membranes, Electrocatalysis, and Photocatalysis
Source: ACS Appl Nano Mater. 2023 Jan 20;6(3):1620–30. doi: 10.1021/acsanm.2c04537 (PMC9926871; doi:10.1021/acsanm.2c04537)
Supplement: Supplementary file 1 — an2c04537_si_001.pdf [file an2c04537_si_001.pdf]

Supporting Information

Nanoscale Iron Redistribution During Thermochemical Decomposition of $CaTi_{1-x}Fe_xO_{3-\delta}$ Alters the Electrical Transport Pathway: Implications for Oxygen-Transport Membranes, Electrocatalysis, and Photocatalysis

Jason Luong[1], Xin Wang[1], Alicia Tsung[2], Nicholas Humphrey[2], Huiming Guo[1], Benjamin X. Lam[1], Shaama Mallikarjun Sharada[2,3], William J. Bowman[1,4,*]

[1] Department of Materials Science and Engineering, University of California, Irvine, Irvine, CA, 92697, USA
[2] Mork Family Department of Chemical Engineering and Materials Science, University of Southern California, Los Angeles CA, 90089, USA
[3] Department of Chemistry, University of Southern California, Los Angeles CA, 90089, USA
[4] Irvine Materials Research Institute, University of California, Irvine, Irvine, CA, 92697, USA

* will.bowman@uci.edu

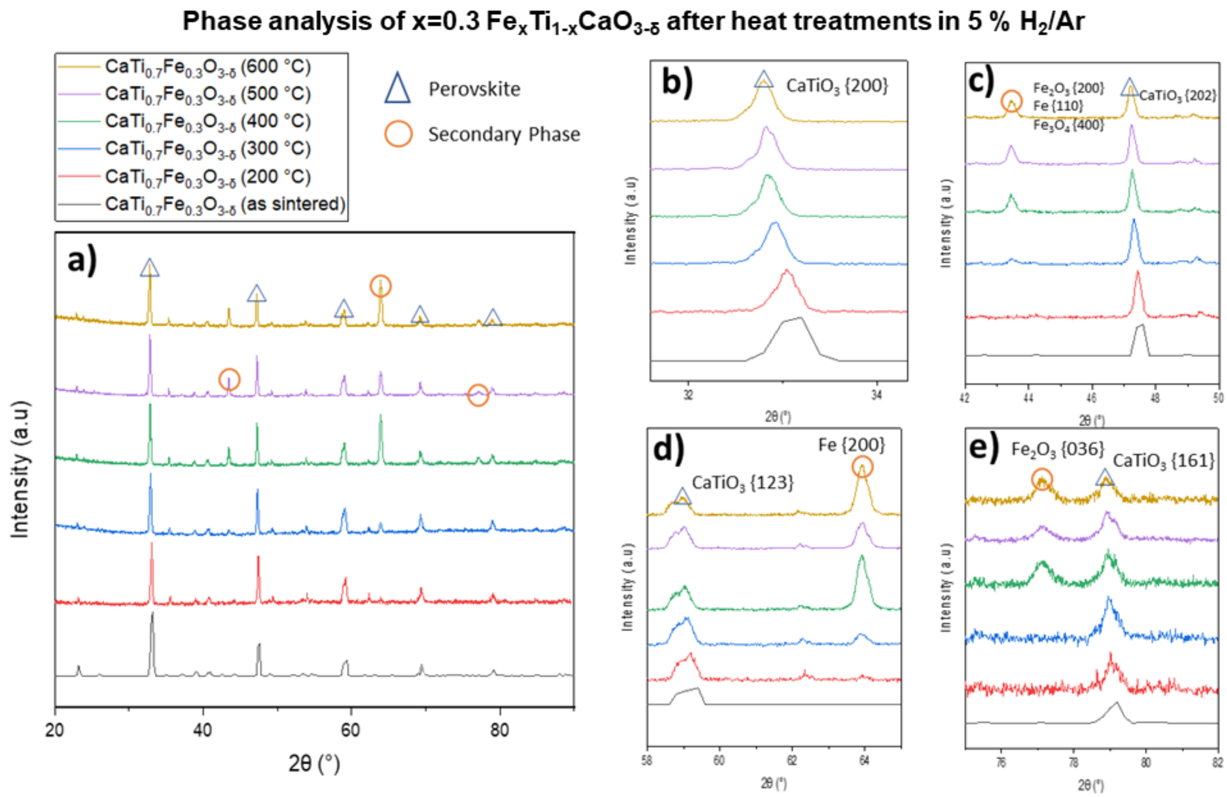

Fig. S1. The XRD patterns of $CaTi_{0.7}Fe_{0.3}O_{3-\delta}$ after undergoing thermochemical treatments of 200-600 °C. (a) XRD pattern with indication of peaks being perovskite phase or secondary phase (marked by triangles or circles respectively). (b) The expanded view of the $CaTiO_3${200} diffraction peak in the range of 32° - 34°. (c) The expanded view of the Fe {110}, Fe_2O_3 {200}, or Fe_3O_4 {400} diffraction peaks in the range of 42° - 50°. (d) The expanded view of the Fe {200} diffraction peak in the range of 58° - 65°. (e) The expanded view of the Fe_2O_3 {036} diffraction peak in the range of 75° - 82°.

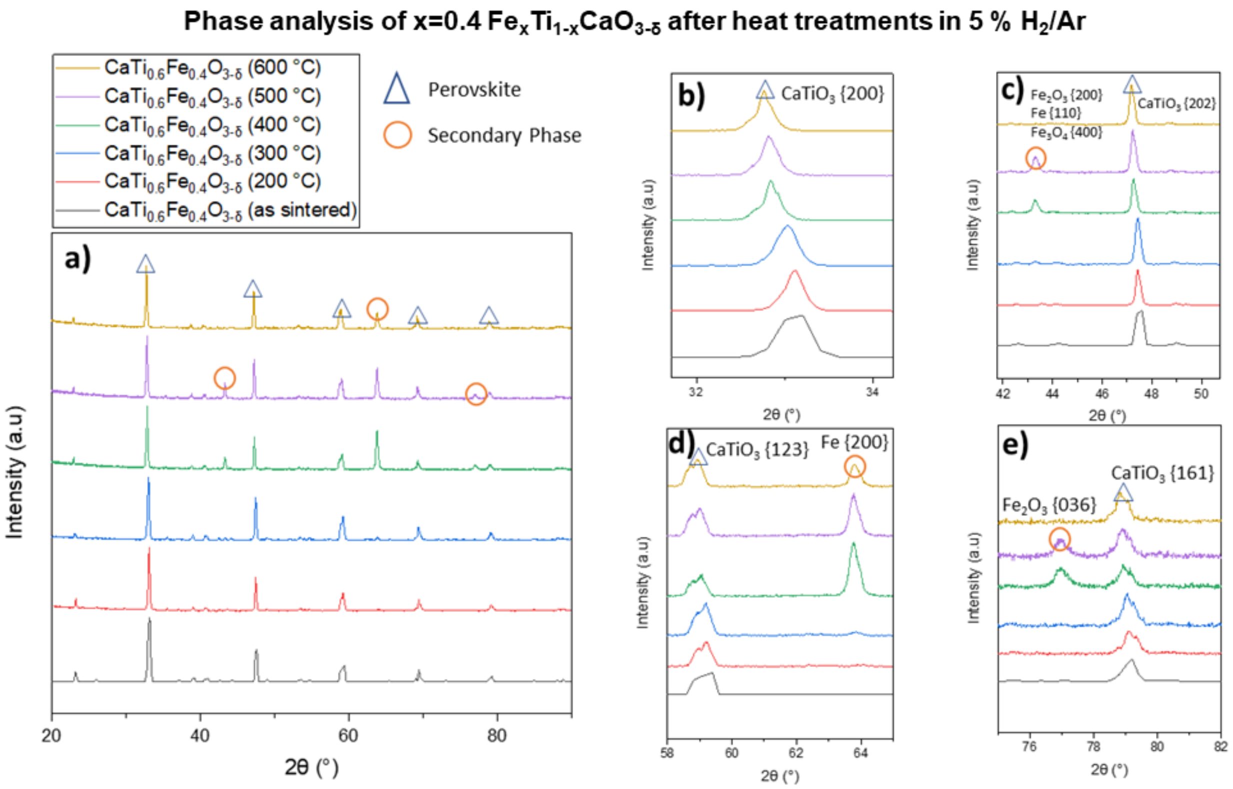

Fig. S2. The XRD patterns of $CaTi_{0.6}Fe_{0.4}O_{3-\delta}$ after undergoing thermochemical treatments of 200-600°C. (a) XRD pattern with indication of peaks being perovskite phase or secondary phase (marked by triangles or circles respectively). (b) The expanded view of the $CaTiO_3${200} diffraction peak in the range of 32° - 34°. (c) The expanded view of the Fe {110}, Fe_2O_3 {200}, or Fe_3O_4 {400} diffraction peaks in the range of 42° - 50°. (d) The expanded view of the Fe {200} diffraction peak in the range of 58° - 65°. (e) The expanded view of the Fe_2O_3 {036} diffraction peak in the range of 75° - 82°.

Table S1. Estimated perovskite matrix unit cell volume changes in x = 0.3 and x = 0.4 $CaTi_{1-x}Fe_xO_3$ based on the {200} XRD peak in Fig. S1b and S2b. Heat treatment temperatures identify samples. * From ICDD database #00-042-0423.

Sample	2θ (°)	d_{200} (nm)	d_{100} (nm)	V_{Cell} (nm^3)	% change
x = 0.3					
As sintered	33.09	0.272 *	0.544	0.161	0.0
200 °C	33.00	0.273	0.545	0.162	0.8
300 °C	32.91	0.273	0.547	0.164	1.6
400 °C	32.83	0.274	0.548	0.165	2.4
500 °C	32.83	0.274	0.548	0.165	2.4
600 °C	32.76	0.275	0.549	0.166	3.0
x = 0.4					
As sintered	33.16	0.272 *	0.544	0.161	0.0
200 °C	33.08	0.272	0.544	0.161	0.1
300 °C	33.02	0.273	0.545	0.162	0.7
400 °C	32.88	0.274	0.547	0.164	1.9
500 °C	32.82	0.274	0.549	0.165	2.5
600 °C	32.71	0.275	0.550	0.167	3.5

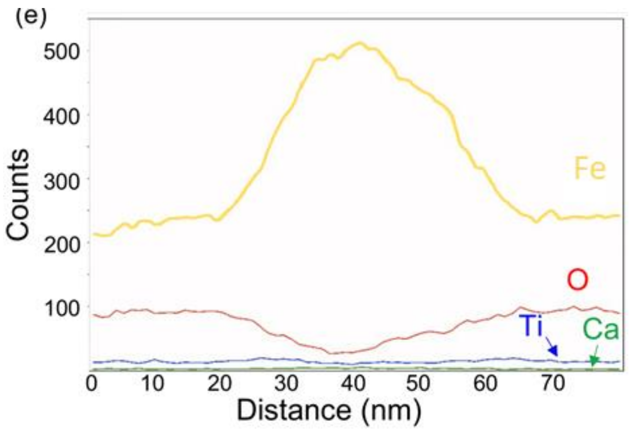

Fig. S3. EDX elemental signal profile along the line in Fig. 3i.

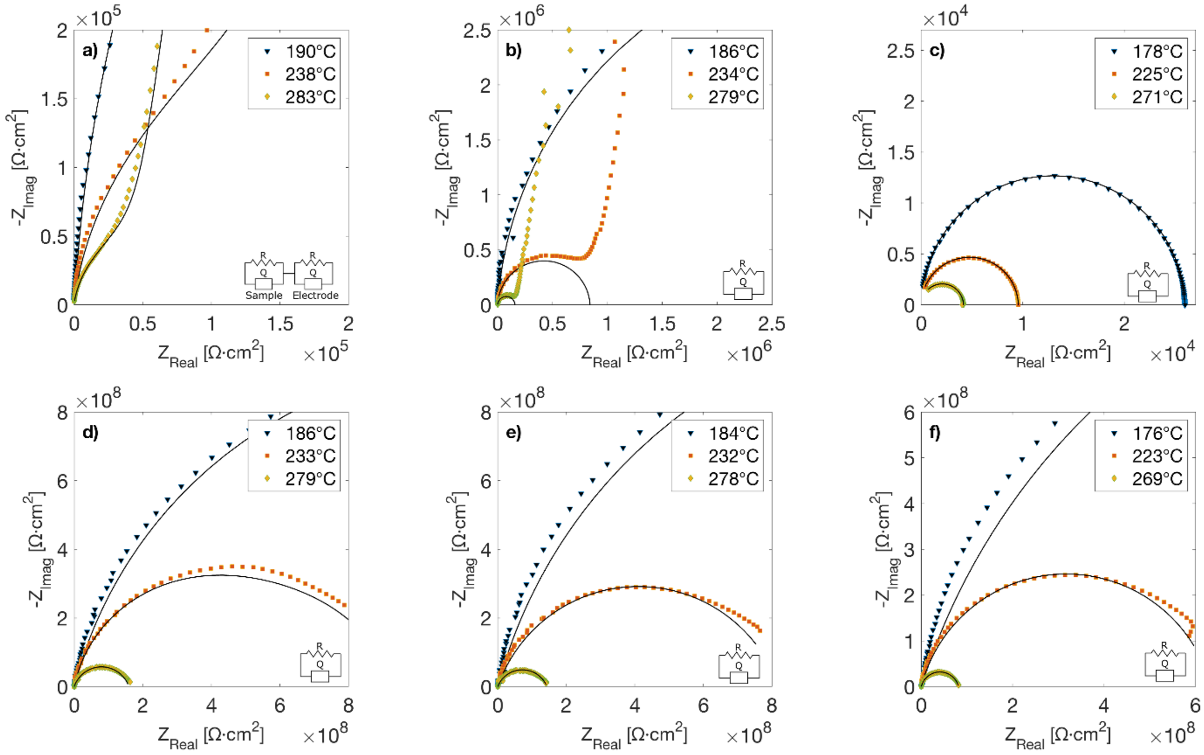

Fig. S4. Representative EIS measurements for all samples across three temperatures for (a) $CaTi_{0.9}Fe_{0.1}O_{3-\delta}$, (b) $CaTi_{0.8}Fe_{0.2}O_{3-\delta}$, and (c) $CaTi_{0.5}Fe_{0.5}O_{3-\delta}$ before thermochemical treatment. Subplots (d, e, f) represent $CaTi_{0.7}Fe_{0.3}O_{3-\delta}$ measured after thermochemical treatment at (d) 400 °C, (e) 500 °C, and (f) 600 °C. The solid lines are equivalent circuit model fitting to the measured data. The highest frequency arc was interpreted as the sample's impedance contribution in cases fit to multiple arcs.

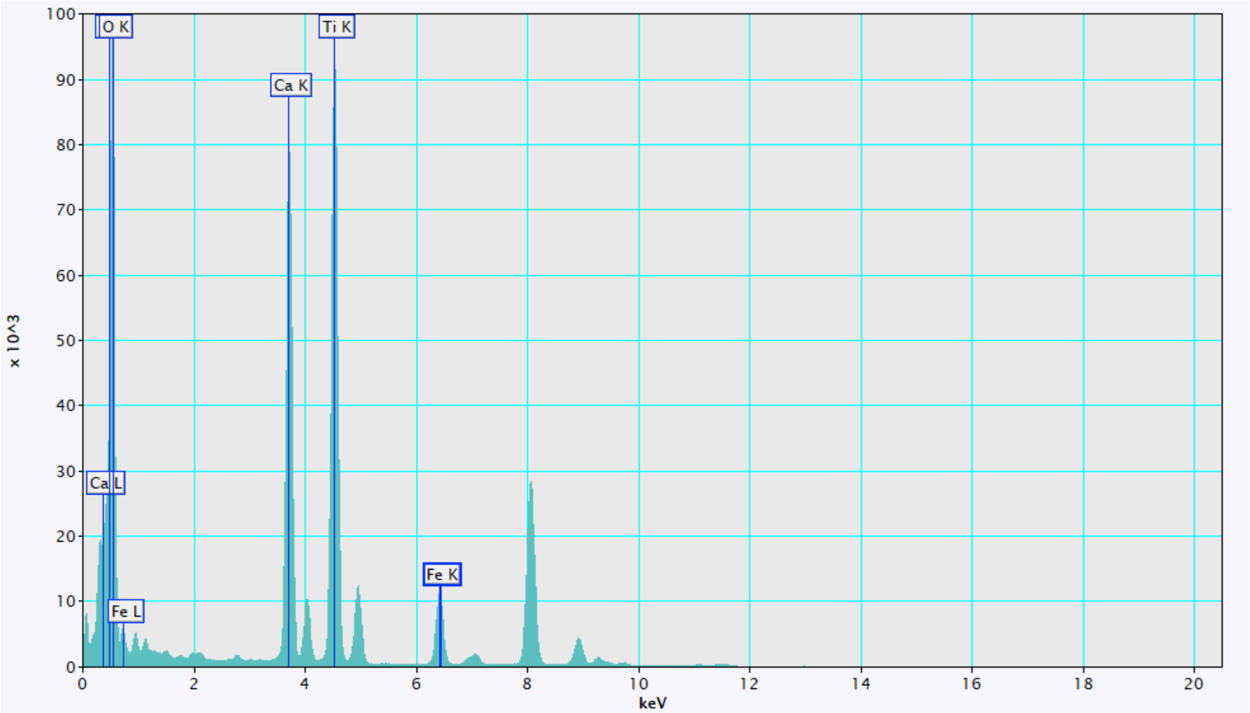

Fig. S5. STEM EDS data summed over all pixels in the area mapped in Fig. 4c. The Fe K, Ca K, Ti K, and O K lines were used to create the atomic-resolution elemental maps in Fig. 4f.
